# Integrated widely targeted metabolomics and network pharmacology revealed quality disparities between Guizhou and conventional producing areas of Codonopsis Radix

**DOI:** 10.3389/fnut.2023.1271817

**Published:** 2023-10-17

**Authors:** Kaixian Zhang, Delin Zhang, Qingfang Yang, Langtao Long, Jian Xie, Yong Wang, Qiuyang Yao, Faming Wu, Sha Liu

**Affiliations:** ^1^School of Pharmacy, Zunyi Medical University, Zunyi, China; ^2^Pharmacy Department, Affiliated Hospital of Zunyi Medical University, Zunyi, China; ^3^Guizhou Medical and Health Industry Research Institute, Zunyi, China; ^4^State Key Laboratory of Functions and Applications of Medicinal Plants, Guizhou Medical University, Guiyang, China

**Keywords:** Codonopsis Radix, DNA barcoding, widely targeted metabolomics, network pharmacology, quality

## Abstract

**Introduction:**

With the internationalization of traditional Chinese medicine, the demand for medicinal and edible Codonopsis Radix (CR) has increased, and its medicinal resources have attracted attention. CR is a well-known traditional Chinese medicine with a long pharmaceutical and edible history. The Guizhou province in China has abundant CR resources, but in the absence of systematic studies on species identification and chemical compositions, the capacity of the capacity of the province to CR resource has not been fully utilized.

**Methodology:**

We used plant morphology and DNA barcoding techniques to identify Luodang (LD) and Weidang (WD) species. To investigate the differences in metabolites between LD and WD, as well as three Chinese Pharmacopeia CRs, and to predict pharmacological mechanisms of action for the dominant differential metabolites, we utilized widely targeted metabolomics and network pharmacology. The results also revealed the material basis for the excellent food properties of both LD and WD.

**Results:**

The plant traits and DNA barcoding molecular identification results indicated that Luodang and Weidang from Guizhou were *Codonopsis tangshen* and *Codonopsis pilosula*, respectively. Widely targeted metabolomics analysis revealed that a total of 1,116 metabolites from 14 categories, including phenolic acids, lipids, flavonoids, were found in five CRs and shared 1,054 (94.4%) metabolites. LD and WD each contained 3 and 10 dominant differential metabolites, respectively, which were primarily flavonoids and amino acids. Amino acids, phenolic acids, and organic acids play important roles in their excellent food attributes. In CR, eight dominant differential metabolites were discovered for the first time, including isoorientin-7-O-(6″-feruloyl) glucoside, N-formyl-L-methionine, and cyclo (Phe-Glu), among others. Network pharmacology analyses showed that, in LD, dominant differential metabolites were closely related to anti-tumor, cardiovascular disease improvement, nervous system protection, and metabolic disease treatment, whereas in WD, they were closely related to nervous system protection and cardiovascular disease improvement.

**Conclusion:**

The species of LD and WD were included in the Chinese Pharmacopeia, and their metabolite profiles were remarkably similar to CR from traditional producing areas. Therefore, LD and WD can be used and promoted medicinally as CR, and they have potential value for new drug development. This study enriched the database of CR compounds and provided a reference for quality control, resource development, and new drug development of CR.

## 1. Introduction

Codonopsis Radix (CR) is a perennial herbaceous dicotyledonous plant of the genus *Codonopsis* belonging to the family Campanulaceae (1). The genus *Codonopsis* contains approximately 40 species that are found throughout eastern and central Asia, including 39 species in China (2). The Chinese Pharmacopeia (2020 edition) defines CR as the dried root of *Codonopsis pilosula* (Franch.) Nannf., *Codonopsis tangshen* Oliv., or *Codonopsis pilosula* Nannf.var.*modesta* (Nannf.) L.T.Shen of the family Campanulaceae (1). They are the most widely used species, and their fresh and dried roots are regarded as famous among traditional Chinese medicines (TCM) with long traditional use (from the Qing Dynasty) (3). CR is known as “Dangshen” in Chinese and “Tojin” in Japanese and has been utilized in Chinese medicine to treat *qi* (vital energy) deficit, boost immunity, treat stomach ulcers, increase appetite, lower blood pressure, and other conditions. It is occasionally used as a ginseng alternative (*Panax ginseng* C. A. Mey.) (4–6). To date, more than 230 compounds have been isolated and identified from CR, including alkaloids, terpenoids, flavonoids, lignans, steroids, and sugars. Among them, alkaloids, polyacetylenes, phenylpropanoids, flavonoids, and polysaccharides are considered to be the main active components of CR (7, 8).

CR is a medicinal material for both medicine and food issued by the Health Commission of China within a limited range and dose (9). According to a total of 602 survey reports on the consumption of CR in 31 provincial-level administrative regions in China, 64.95% of respondents reported that they had eaten CR, while 86.19% of respondents described that people around them had the habit of eating CR, with an annual edible amount of 8,000–10,000 tons (10). The usual methods of consuming CR include making soup, tea, wine, and porridge with it. In addition, CR is also consumed in Singapore, Malaysia, Australia, Thailand, Korea, Japan, North Korea, and other countries; especially in Singapore, Malaysia, and other Southeast Asian countries, CR is used as an ingredient for healthcare in their daily diet (10). According to the report on the TCM circulation market released by the Chinese Ministry of Commerce in 2017, the export value of CR ranked ninth among all exported TCMs, with an export volume of 3,452.49 tons (11).

CR is distributed throughout China and mainly produced in the southwestern provinces (2). The main producing areas of *C. pilosula, C. pilosula* var. *modesta*, and *C. tangshen* are Jincheng (Shanxi province) and Weiyuan (Gansu province), Jiuzhaigou (Sichuan province) and Weiyuan (Gansu province), and Wuxi (Chongqing) and Enshi (Hubei province), respectively (12). Unfortunately, despite having the most abundant CR resources, Guizhou province is not the main producing area of CR, and the main reason is the lack of basic research on species identification and chemical compositions. Owingue to Guizhou's unique climatic conditions and location advantages, Luodang (LD), produced in Daochen county, and Weidang (WD), produced in Weining county, have excellent edible characteristics such as good taste, easy slagging, and no wood core, which are best among traditional edible CR and loved by the majority of diners. In addition to catering to domestic demand, LD and WD are also exported to a large number of Southeast Asian countries and others as well (13).

At present, details on the species of LD and WD are still limited. Studies have reported that LD is an ecological variant of *C. tangshen*, but there is no strong evidence to support this claim, and there is no information on the study of the origin of WD. The lack of species identification research has greatly hindered the development and utilization of LD and WD, which needs to be urgently addressed (14). The common and simple methods to address this problem are plant trait characterization and DNA barcoding molecular identification (15). DNA barcoding is a molecular technique that involves obtaining short DNA sequences from standardized regions of the genome for species identification (16, 17). DNA barcoding regions can be detected in all tissues of plants regardless of factors such as growth, development, differentiation, or environment (18). Among them, ribosomal DNA internal transcribed spacer (ITS) sequences have been widely used for plant species identification and phylogenetic relationship analysis due to their conserved length, abundant informative sites, and rapid nucleotide variation (19–21). In recent years, there have been numerous studies on molecular identification, genetic diversity, and phylogenetic classification of medicinal plants (22–24). For example, researchers used ITS (25), ITS2 (9, 25, 26), and *psb*A-*trn*H (9) barcodes to distinguish CR and its related species and achieved better results. CR is a TCM with multiple species and producing areas, and the species and producing areas have significant effects on the accumulation of metabolites. Currently, by utilizing the high-throughput and unbiased comprehensive knowledge of widely targeted metabolomics, it is possible to qualitatively and quantitatively analyze small molecule metabolites from different sources of plants and identify the law of metabolic changes. Metabolomics research has become a hot research topic in the post-genomic era. It has been widely applied to the study of secondary metabolites in medicinal plants such as *Dendrobium officinale* Kimura et Migo (27), *Pueraria lobata* (Willd.) Ohwi (28, 29), *Curcuma Longa* L. (30), *Lycium barbarum* L. (31), *Scutellaria baicalensis* Georgi (32), and *Sesamum indicum* L. (33). In contrast, studies on the metabolomics of CR have only been conducted on the traditional producing areas, while few studies have been reported on non-traditional producing areas such as Guizhou. Therefore, it is crucial to determine the specificities of the LD and WD species, explore the differences in metabolite levels between them and CR from traditional producing areas, and identify the material basis of their excellent edible characteristics. These aspects will aid further in their in-depth development, utilization, and formulation of standardized quality measures.

Plant morphology and DNA barcoding molecular identity were merged in this study to distinguish LD and WD and explain their species. Furthermore, the metabolite differences between LD and WD, as well as the traditional CR-producing areas, were investigated using widely targeted metabolomics and network pharmacology analysis. The dominant differential metabolites of LD and WD were screened, the pharmacological mechanisms of action of the dominant differential metabolites were predicted, and their medicinal properties were analyzed. This study will aid in understanding the species information, metabolite characteristics, and pharmacological action mechanisms of LD and WD. It also aims to provide references for expanding the medicinal source of CR, improving quality standards, developing and utilizing LD and WD resources, and researching new drugs.

## 2. Materials and methods

### 2.1. Sample collection

A total of fifteen samples were collected from August 2021 to October 2021 at the beginning of our research group. LD and WD samples were collected from Daozhen county and Weining county of Guizhou province, respectively. *C. pilosula* (CP) and *C. pilosula* var. *modesta* (CPM) were collected from Weiyuan county and Wenxian county of Gansu province, respectively. *C. tangshen* (CT) of collected from Wuxi county of Chongqing city (Table 1). The amount of each sample ranged from 1,000 to 2,000 g, and three samples were collected from each region. Samples were immediately transported to the laboratory, rinsed, and divided into aboveground and underground portions that were sealed in self-sealing bags. The aboveground portion was kept at −20°C for species identification, while the underground portion was kept at −80°C for metabolite extraction. The CP, CPM, and CT samples were identified by Professor Faming Wu (College of Pharmacy, Zunyi Medical University) as the roots of *C. pilosula, C. pilosula* var. *modesta*, and *C. tangshen*, respectively.

**Table 1:** Detailed information of Codonopsis Radix.

**Sample name**	**Producing area**	**Longitude**	**Latitude**	**Number of sample**
CP *(C. pilosula)*	Weiyuan, Gansu	104°20′6″	35°5′7″	3
CPM (*C. pilosula* var. *modesta*)	Wenxian, Gansu	104°35′24″	33°8′44″	3
CT (*C. tangshen*)	Wuxi, Chongqing	109°29′38″	31°24′32″	3
LD	Daozhen, Guizhou	107°43′25″	28°0′47″	3
WD	Weining, Guizhou	103°51′45″	26°35′21″	3

### 2.2. Species identification

Observations on the stems, leaves, and flowers of plant specimens of CR were checked according to the Pharmacopeia of the People's Republic of China (2020 edition) and the *Flora of China* (vol. 73, sub vol. 2). Upon combining information from the origin survey, we initially identified the species of LD and WD.

The DNA barcoding molecular markers were applied to identified five species of CR according to the method described by Li et al. (9). After being thoroughly ground in liquid nitrogen with the proper quantity of fresh samples from the CR's aboveground part, the DNA from the samples was extracted using a DNA extraction kit [Sangon Biotech (Shanghai) Co., Ltd.]. The ITS2 amplification primers were ITS 2F: 5′-ATGCGATACTTGGTGTGAAT-3′ and ITS 3R: 5′-GACGCTCTCCAGACTACAAT-3′. The primers were synthesized by Shanghai Bioengineering Co., Ltd., and the sample DNA was used as the template for PCR amplification of ITS2 sequences. Polymerase chain reaction (PCR) was performed according to the instructions of the PCR reaction kit [Sangon Biotech (Shanghai) Co., Ltd.]. PCR reaction conditions included pre-denaturation at 94°C for 5 min; denaturation at 94°C for 30 s, annealing at 56°C for 30 s, extension at 72°C for 45 s (35 cycles); extension at 72°C for 10 min, and termination storage temperature of 4°C. Using a pipette, 2.5 ml of PCR amplification product and 6 × loading buffer were added to the wells of a prepared 1.2% agarose gel. The agarose gel was placed in an electrophoresis tank for electrophoresis at 120 V, 80 mA for 35 min, after which it was taken out at the end of the electrophoresis, and the PCR products were sequenced by Sangon Biotech (Shanghai) Co., Ltd.

The standard ITS2 spacer sequence was obtained by excising the 5.8S and 28S regions at both ends of the sequence using the ITS2 database (http://its2.bioapps.biozentrum.uni-wuerzburg.de/). The resulting sequence was sequenced by the Blast database (https://blast.ncbi.nlm.nih.gov/Blast.cgi) for comparison, and the relevant reference sequences were downloaded based on the following comparison results: MF096135, MF096136, MF096142, MF096143, MF096146, MF096147, MF096150, KP318239, KP318243, KP318249, DQ889459, and AF136237. The Mega11.0 software was used to construct a phylogenetic tree using the neighbor-joining (NJ) method. The support of each branch of the clustering tree was examined using a bootstrap test (Bootstrap) with a self-expansion set to 1,000 iterations. The plant morphology and DNA barcode molecular identification methods were combined to identify the CR species.

### 2.3. Widely targeted metabolomic profiling

#### 2.3.1. Sample preparation

CR samples were prepared by reference methods (27). Fresh CR samples were vacuum freeze-dried in a freeze-dryer (Scientz-100F, Scientz, China) before being crushed to powder in a blender (30 Hz, 1.5 min, MM 400, Retsch, Germany). Approximately 50 mg of the sample was weighed, dissolved in 1.2 mL of 70% methanol (Merck, Germany) extract, and vortexed (IKA VORTEX GENIUS 3, VG 3 S25) every 30 min for 30 s for a total of 6 times. The sample was placed at 4°C overnight and centrifuged at 12,000 rpm for 10 min, and then, a 0.22-μm microporous membrane was used to filter the supernatant, which was then placed in a vial for an ultra performance liquid chromatography-electrospray ionization-tandem mass spectrometry (UPLC-ESI-MS/MS) analysis.

#### 2.3.2. UPLC-ESI-MS/MS analysis

The CR extracts were analyzed by widely targeted metabolomics using UPLC-ESI-MS/MS (34). Chromatographic separation was done using an Agilent SB-C18 column (1.8 m, 2.1 mm, 100 mm; Agilent, Foster City, CA, USA) on a Nexera X2 UPLC machine (Shimadzu, Kyoto, Japan). Purified water with 0.1% formic acid (solvent A) and acetonitrile with 0.1% formic acid (solvent B) made up the mobile phase. The gradient elution program was as follows: 0–9 min (95% → 5% A), 9–10 min (5% A), 10–11.1 min (5% → 95% A), and 11.1–14 min (95% A). The flow rate was 0.35 mL/min, the injection volume was 2 μL, and the temperature in the column oven was set at 40°C. The effluent was alternatively introduced into a triple quadrupole-linear ion trap mass spectrometer (6500 Q-TRAP UPLC–MS/MS; Applied Biosystems, Framingham, MA, USA) equipped with an ESI Turbo Ion-Spray interface. The following settings were used to run the ESI ion source: ion source, turbo spray; temperature, 550°C; spray voltage, 5,500 V (positive ion mode)/-4,500 V (negative ion mode); gas I, 50 psi; gas II, 60 psi; curtain gas, 25.0 psi; and collision-activated dissociation, high. Instrument calibration and mass calibration were carried out with 10 and 100 mol/L polypropylene glycol solutions in triple quadrupole and linear ion trap modes, respectively. The multiple reaction monitoring (MRM) assays were used to collect triple quadrupole images with the collision gas (nitrogen) set to medium. The delustering potential and collision energy were optimized for individual MRM transitions to enhance their efficiency. Each period was examined for a specified set of MRM transitions based on the metabolites eluted during that interval. Analyst v1.6.3 (AB Sciex, Foster City, CA, USA) was used to collect and analyze mass spectra.

The resultant mass spectra were compared in the Metware Biotechnology Co., Ltd. (MWDB) database. Interference signals, such as the repeated signals of K^+^, Na^+^, NH^4+^ ions, the repetitive signals of fragment ions, and the isotope signal were initially eliminated throughout analyses to assure the correctness of the metabolite annotations. MRM-based quantification of metabolites was conducted using Multi Quant v3.0.2 (AB Sciex). After obtaining the signal intensities of each metabolite's distinctive ions in several samples, the chromatographic peak regions of those ions were integrated to indicate the metabolite's relative abundance. The peak areas were adjusted with retention time and peak type to make it easier to compare the same metabolites quantitatively between samples.

### 2.4. Network pharmacology

The Canonical SMILES of the dominant differential metabolites were searched using the PubChem database (https://pubchem.ncbi.nlm.nih.gov/) and then entered into the Swiss Target Prediction database (http://www.swisstargetprediction.ch/) for the prediction of corresponding target proteins (35). The targets of the dominant differential metabolites were identified after the relevant target proteins had been sorted and the repetitive targets had been eliminated.

A network was constructed and visualized with default parameters and similarity thresholds using STRING (http://string-db.Org, high confidence > 0.9) and Cytoscape v3.7.1. Gene Ontology (GO, http://geneontology.org/) functional enrichment, Kyoto Encyclopedia of Genes and Genomes (KEGG, https://www.genome.jp/kegg/) pathway enrichment, and disease enrichment analyses were performed using the DAVID database (https://david.ncifcrf.gov/) (*p* < 0.01).

### 2.5. Statistical analysis

Raw data were organized with Microsoft Office Excel 365. The R software version 4.2.3 (http://www.r-project.org) was used to perform principal component analysis (PCA), hierarchical cluster analysis (HCA), orthogonal partial least squares-discriminant analysis (OPLS-DA), and graphing. Metabolites with variable importance in the projection (VIP, OPLS-DA) and fold change ≥2 or fold change ≤ 0.5 were considered differential metabolites (DMs) (36).

## 3. Results and discussion

### 3.1. Species identification

**Figure 1:**
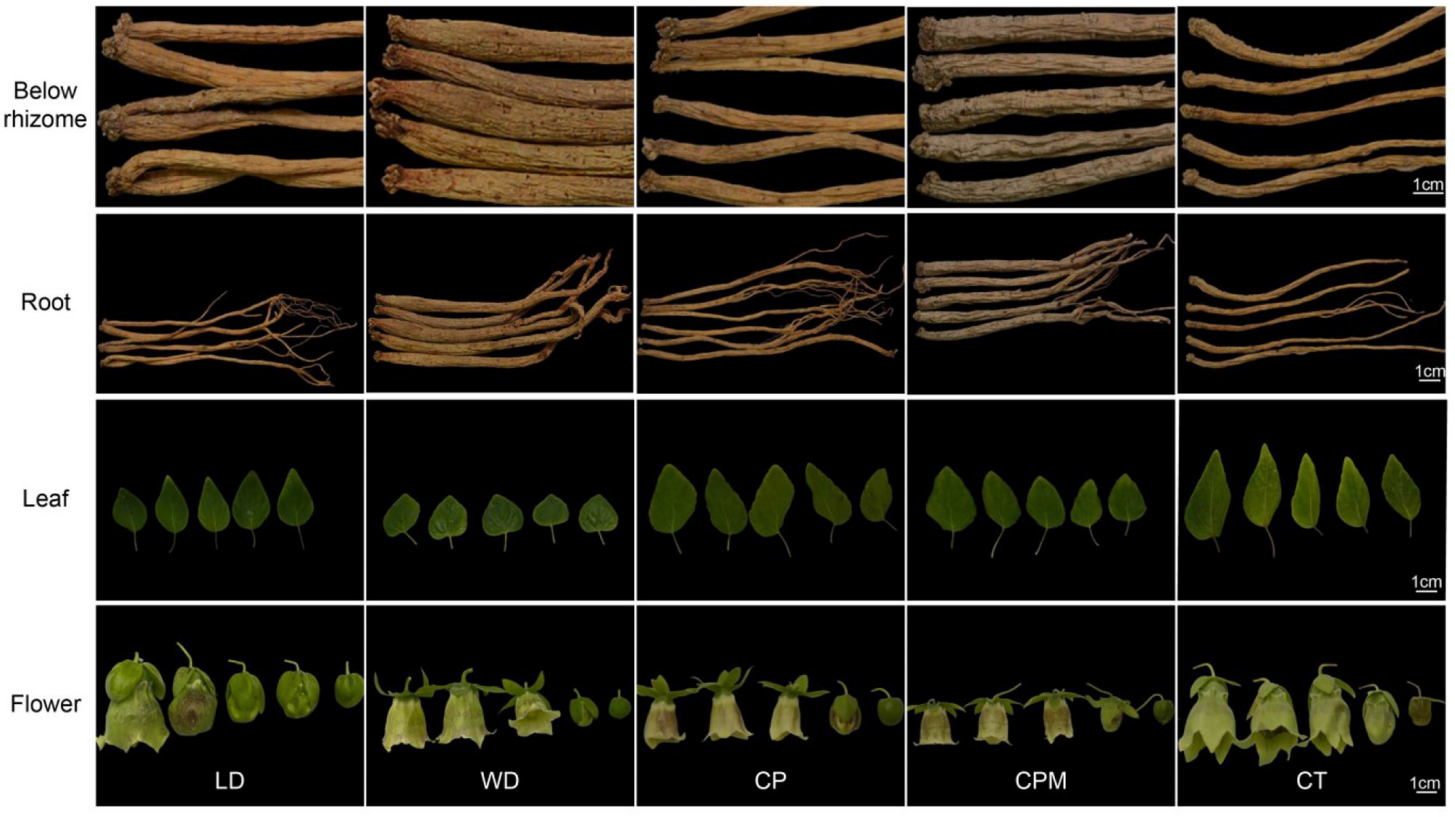
Morphological characteristics of Codonopsis Radix.

We observed the appearance traits of the following five species of CR: *C. pilosula, C. pilosula* var. *modesta, C. tangshen*, LD, and WD. We found that the dried root surfaces of LD and WD had no obvious transverse lines, the same as those of *C. pilosula* and *C. tangshen* in the Chinese Pharmacopeia; however, there was a significant difference with the dense ring striation under the root head of *C. pilosula* var. *modesta*. The corolla of the five species of CR was campanulate or tubular-campanulate, with the calyx lobes entire, without obvious differences. On the other hand, LD's leaf blade was narrowly ovate or lanceolate, blunt or acute at the tip, cuneate or more rounded at the base. Similarly, WD's leaf blade was ovate or narrowly ovate but subcordate at the base and hairy. The leaf characteristics of LD and WD were similar to those of *C. pilosula* and *C. tanshen* (recorded in the *Flora of China*), respectively (Figure 1).

**Figure 2:**
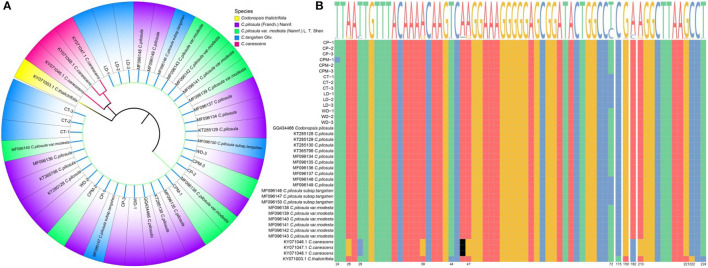
Construction of NJ tree of Codonopsis Radix samples based on ITS2 sequences **(A)**. ITS2 sequence variant site information **(B)**. LD, WD, CP, CPM, and CT reported Luodang, Weidang, *C. pilosula, C. pilosula* var. *modesta*, and *C. tangshen*, the same below.

The ITS2 sequence length of *C. pilosula, C. pilosula* var. *modesta, C. tangshen, Codonopsis thalictrifolia*, LD, and WD was 239 bp, while the ITS2 sequence length of *Codonopsis canescens* was 238 bp. The guanine-cytosine (GC) content was 61.09%−63.87%, and the high GC content indicated that the DNA double-strand was stable and could be used as an auxiliary indicator for the identification of the above seven CR species (the five species of CR along with LD and WD). To eliminate individual differences among samples, we performed multiple sequence comparisons and calculated the average Kimura 2-parameter (K2P) distances among CR species separately. The results showed that the intraspecific and interspecific variation sites were 2 and 14, respectively; genetic distance: intraspecific (0)> interspecific (0.000–0.020). The K2P distances between *C. pilosula*, and *C. pilosula* var. *modesta* was 0.000, whereas those between *C. tangshen* and *C. pilosula, C. pilosula* var. *modesta* were 0.010, showing a tighter genetic affinity between *C. pilosula* and *C. pilosula* var. *modesta*. The neighbor-joining (NJ) clustering tree of ITS2 sequences clustered the seven CR sequences into three categories (Figure 2), with *C. pilosula, C. pilosula* var. *modesta, C. Pletangshen, C. thalictrifolia*, LD, and WD clustered into one category, and *C. canescens* and *C. thalictrifolia* each clustered into a separate category. Combined with the result of plant traits and DNA barcoding molecular identification, we assumed that LD was *C. tangshen*, and WD was *C. pilosula*.

The genus *Codonopsis* is widely distributed in China, but only three species (3/39, 7.7%) are included in the Chinese Pharmacopeia as legal species for medicinal or food use throughout the country (1). Guizhou is rich in species of the genus *Codonopsis*—eight species and five varieties have been identified, accounting for approximately one-third of the total number of the genus (37). Previous studies have found that *Codonopsis* has the characteristic of secondary pollen display, and its dioecious and dioecious characteristics effectively avoid self-pollen interference. This is a typical heterozygous breeding system with no pollen limitation for fruiting and seed set, but this characteristic is very likely to cause interspecific and intraspecific hybridization during the introduction of *Codonopsis* (38). Most of the commercially available medicinal CR herbs are *C. pilosula*, and the artificial cultivation of *C. pilosula* is often simple domestication or introduction of wild species from local seed sources. The mixed cultivation of different species of *C. pilosula* or groups with large differences in genetic traits has resulted in confusion about the species of *C. pilosula* herbs and their uneven quality. It is difficult to effectively control its quality, which directly affects the clinical use of Chinese medicine. This has a direct effect on the safety and efficacy of the clinical use of Chinese medicine and severely restricts the sustainable development of the CR industry (29). Therefore, the correct identification of CR herbs and their closely related species and mixed fakes is crucial.

Our study found that LD and WD from Guizhou were *C. tangshen* and *C. pilosula*, respectively. Unexpectedly, the phylogenetic tree constructed based on ITS2 sequences was able to distinguish *C. pilosula* (CP), *C. pilosula* var. *modesta* (CPM), and *C. tangshen* (CT) from other species of the genus *Codonopsis* but could not establish their separation. The reason for the unsuccessful identification was that these three species of CR were closely related, and the only difference between *C. pilosula* and *C. pilosula* var. *modesta* was in the two aspects of plant hair and dry root surface circular stripes (1). In 2011, the *Flora of China* (Vol. 19) changed *C. pilosula* var. *modesta* to *C. pilosula* subsp. *pilosula* (former subspecies) and *C. tangshen* was changed to *C. pilosula* subsp. *tangshen*, downgraded to a subspecies of *C. pilosula*, which provided an essential basis for the classification and affinities at the molecular level and also confirmed the correctness of the Chinese Pharmacopeia to include the three primitive species in the same herbal medicine as CR. Although the DNA barcoding molecular identification method has achieved some success in identifying Chinese herbs and their hybrids, there is still the problem of unsuccessful identification between subspecies, varieties, and original varieties. Follow-up studies might consider addressing this issue at the genomic or proteomic level.

### 3.2. Widely targeted metabolomics analysis

#### 3.2.1. Analysis of differences in metabolite accumulation of five Codonopsis Radix

**Figure 3:**
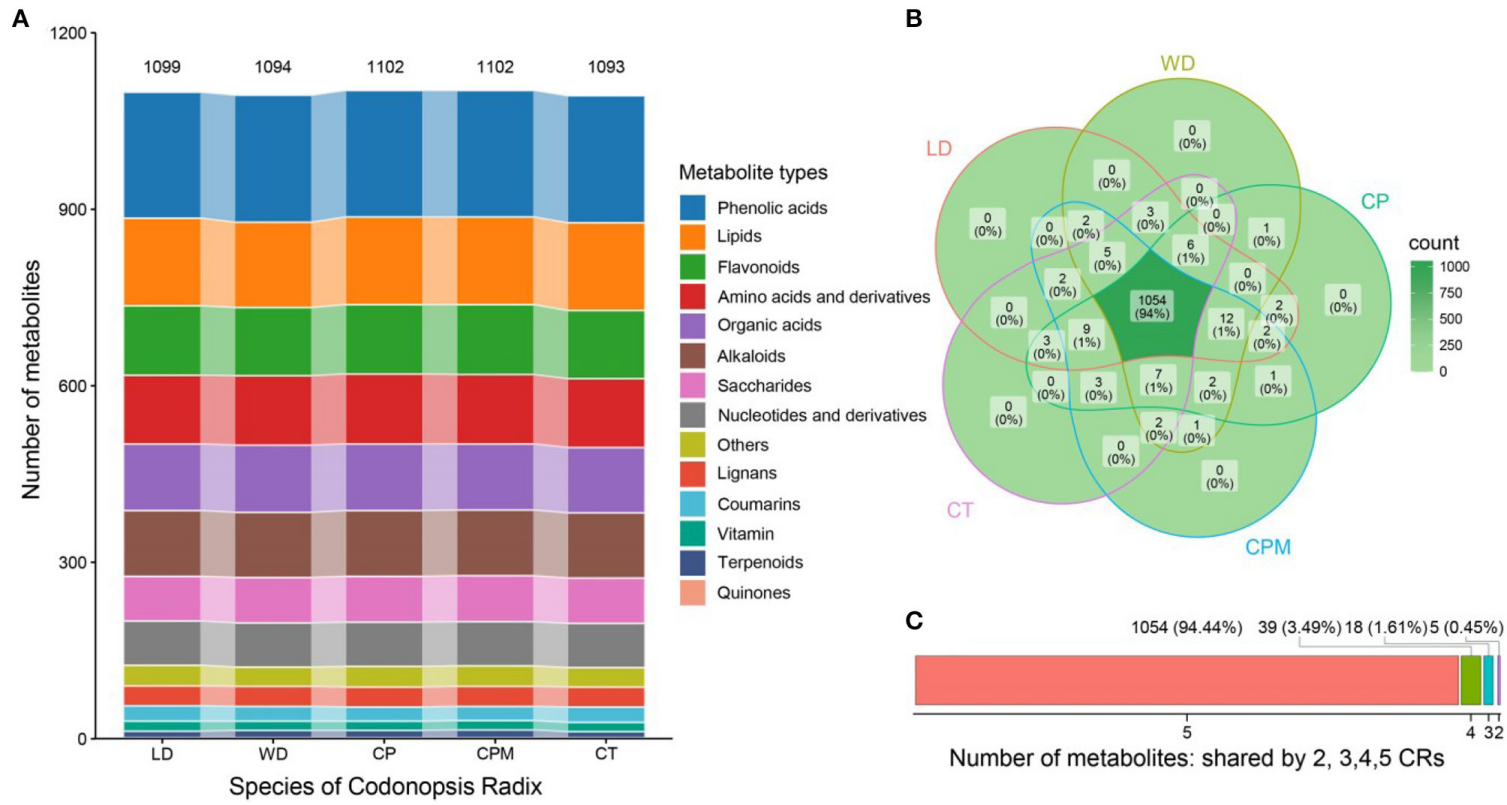
Analysis of differences in metabolite accumulation of five Codonopsis Radix. **(A)** Type distribution of Codonopsis Radix metabolites; **(B)** Codonopsis Radix metabolite venn diagram; **(C)** Number of metabolites shared by Codonopsis Radix. LD, WD, CP, CPM, and CT reported Luodang, Weidang, *C. pilosula, C. pilosula* var. *modesta*, and *C. tangshen*, the same below.

We used UPLC-ESI-MS/MS to analyze the metabolites to further understand the differences in metabolite accumulation of the five species of CR. Approximately 1,116 metabolites were found and identified in the five species of CR, divided into 14 different chemical classes (Figure 3C). The number of metabolites was 1,099 (LD) > 1,094 (WD) > 1,102 (CP) > 1,102 (CPM) > 1,093 (CT). Phenolic acids were the most prevalent class (214–216, 19.3%). The remaining classes were lipids (145–149, 13.3%), flavonoids (116–119, 10.5%), amino acids and their derivatives (117–119, 10.6%), organic acids (111–114, 10.1%), alkaloids (111–112, 10.0%), saccharides (76–78, 6.9%), nucleotides and derivatives (75, 6.7%), Others (33–35, 3.1%), lignans (34, 3.0%), coumarins (24–26, 2.2 %), vitamin (16–17, 1.5 %), terpenoids (10–13, 1.0 %), and quinones (2, 0.2 %) (Figure 3A).

Numerous investigations on the chemical composition of CR have been published thus far. More than 200 different substances had been identified from CR, including polynyas, polyenes, flavonoids, sugars, volatile oils, alkaloids, organic acids, and amino acids (8, 39). Approximately 1,116 compounds were discovered in this study that were based on an earlier study, considerably enhancing the compound database of CR and supplying the scientific basis for the CR quality control study. According to previous reports, phenylpropanoids, alkaloids, flavonoids, and polysaccharides make up the majority of CR's pharmacological constituents. These substances have pharmacological effects on the nervous, endocrine, and immune systems that manifest as anti-tumor, anti-inflammatory, anti-stress, and hepatoprotective effects (8). The metabolomic analysis revealed that the average number of phenylpropanoids in CR, comprising phenolic acids, lignans, and coumarins, was 274.2 (24.6%), accounting for the highest proportion. Phenolic acids are abundant in medicinal plants and have significant medicinal properties that can be made into medications or health foods with antibacterial, anti-inflammatory, analgesic, and anticancer properties. Among them, hydroxybenzoic acid and hydroxycinnamic acid are the main phenolic acids, which are derived from phenylalanine. They are important intermediates in the shikimic acid pathway and participate in the synthesis of subsequent lignans, flavonoids, stilbenes, and alkaloids (40). They are also aromatic secondary metabolites that give typical sensory properties to foods and are associated with a variety of health benefits (41). Our analysis also revealed that there were many kinds of alkaloids (111.6,10.0 %) and flavonoids (117.4,10.5 %) in CR. Modern pharmacological studies have shown that alkaloids and flavonoids have pharmacological effects, such as protecting the nervous system and promoting the circulatory system, in addition to anti-oxidation and anti-fatigue properties (8).

We also found 77.2 (6.9%) different species of saccharides in CR. Among different saccharides, polysaccharides are very significant macromolecules that are crucial for the development and growth of organisms (42). Studies on phytochemistry and biology have revealed that the primary bioactive components of CR are carbohydrates. The main element of CR that has immunomodulatory and anticancer actions is polysaccharide, a significant and comparatively non-toxic natural plant active biological macromolecule (43). At the same time, polysaccharides interact with flavor compounds in the food system, which has an effect on how flavor components are retained and released. CR polysaccharide is an important component affecting the flavor of CR (44). Further, we found high levels of lipids, organic acids, amino acids, vitamins, and other substances in CR. As the basic component of protein and the essential nutrients of the human body, amino acids not only determine the nutritional status of food but also affect the flavor of food and the physiological activity of drugs (45). Amino acids are important flavor substances. Aspartic acid and glutamic acid are umami, and alanine, proline, serine, threonine, and glycine are sweet (46). We found CR to be rich in amino acids (117.2,10.5 %) and a plant protein source with high medicinal value (47). In addition to rich nutrients, CR also has excellent flavor and food attributes related to taste. CR can be further developed and applied in food applications.

Approximately 1,054 metabolites were common to the five CR species, accounting for 94.44%, indicating that their metabolite accumulation characteristics were highly similar (Figure 3B). A total of 39 (3.49 %), 18 (1.61 %), and 5 (0.45 %) metabolites were detected in 4, 3, and 2 species of CR, respectively (Figure 3C). The five species of CR had no unique metabolites. This is because they were all related to the species, subspecies, or varieties of *Codonopsis pilosula* (Franch.) Nannf. in plant taxonomy, and their genetic relationship is very close. This supports the correctness in the *Flora of China* classification of *C. pilosula, C*. pilosula var. *modesta*, and *C. tangshen* as the species of *Codonopsis pilosula* (Franch.) Nannf. and the Chinese Pharmacopeia's classification of these three species as the source of CR. Meanwhile, LD and WD from Guizhou belong to the species included in the Chinese Pharmacopeia in terms of botanical taxonomy, and the chemical composition is highly similar to the traditional genuine producing areas. Therefore, LD and WD can be used and promoted as medicinal herbs. Compared to CP, CPM, and CT, 12, 14, and 18 metabolites were detected only in LD and 13, 10, and 18 metabolites were detected only in WD, respectively (Figure 3B). This might be related to the fact that Guizhou's production area is in a region with low longitude, low latitude, high altitude, heavy precipitation, high annual average temperatures, and average relative humidity. Moreover, both species (29, 48) and the growing environment have a strong influence on the accumulation of secondary metabolites in plants, and metabolite accumulation can vary to some extent between the same species based on their growing environments (27, 31).

The variation in the metabolic characteristics of medicinal plants is the result of the interaction of species, climate, altitude, rainfall, soil, and other environmental factors (32). Under relatively stable conditions, typical genuine medicinal material can be formed. When the habitat is changed or the species are artificially introduced to a different ecological environment, the accumulation of their metabolites will change accordingly; the greater the difference in environmental conditions, the greater their metabolic variation (49). The secondary metabolites of medicinal plants are the material basis of their potency and quality, and studies have confirmed that the quality of CR is significantly affected by species, origin, altitude, latitude, and longitude (50–52). Wu et al. (53) used the Max Ent model and Arc GIS technology to analyze the main environmental factors affecting the distribution of CR herbs by combining 55 environmental factors, and the results showed that precipitation and altitude had major effects on the distribution of all three CR species (*C. pilosula, C. pilosula* var. *modesta*, and *C. tangshen*). In conclusion, species and environmental variables affect CR quality, and these considerations should be completely considered when introducing artificial cultivation to prevent substantial financial losses.

**Figure 4:**
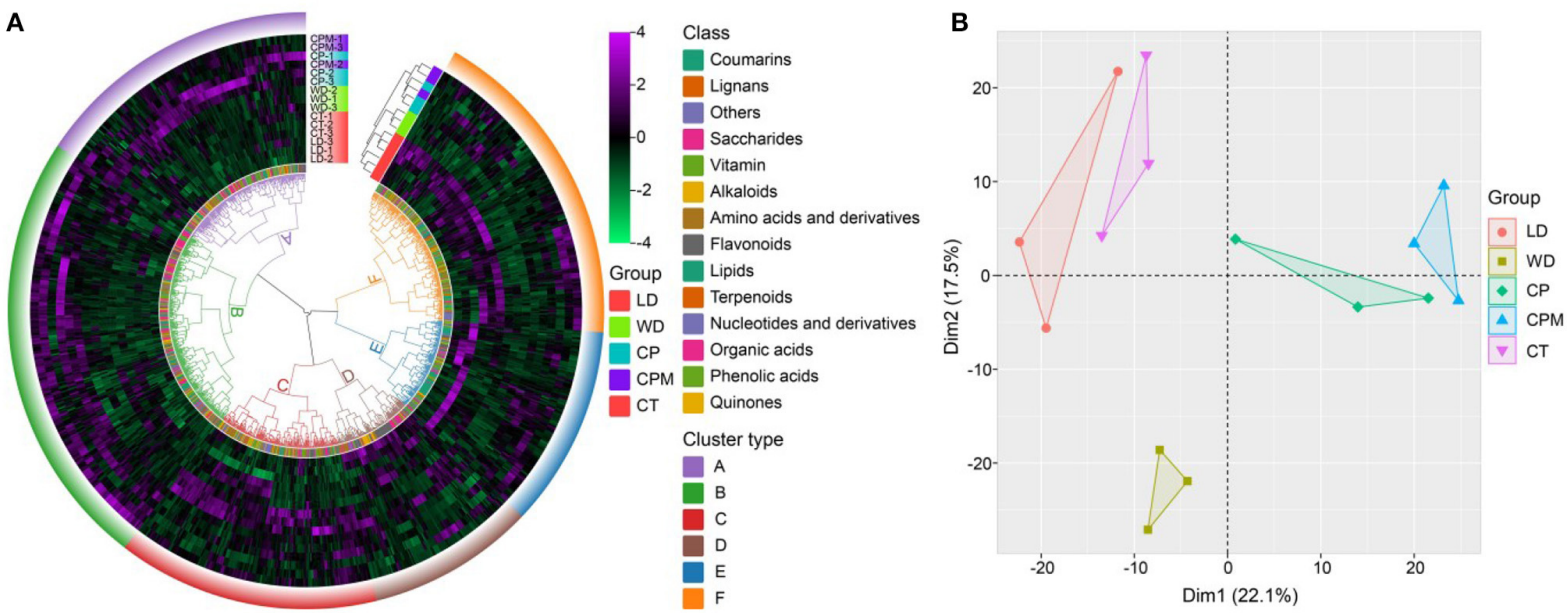
Multivariate statistical analysis of different Codonopsis Radix metabolites. **(A)** Hierarchical cluster analysis of five Codonopsis Radix metabolites; **(B)** Principal component analysis of five Codonopsis Radix metabolites.

To further evaluate the fundamental properties and variations of metabolites between LD and WD and CP, CPM, and CT, we carried out hierarchical cluster analysis and principal component analysis. The results of the cluster analysis revealed that the various CR samples were divided into two categories, where CP and CPM were clustered into one category and CT, LD, and WD were clustered into one category, indicating that CP and CPM metabolites had similar characteristics for accumulation, while CT, LD, and WD metabolites had similar characteristics for accumulation. In terms of metabolites, they were clustered into 6 categories, of which B, C, and E were dominated by phenolic components, accounting for 32%, 17%, and 20%, respectively. A, D, and F were dominated by lipids, amino acids and derivatives, and flavones, accounting for 33%, 28%, and 29%, respectively (Figure 4A). Principal component analysis showed that Dim1 and Dim2 accounted for 22.1% and 17.5% of the total variation, respectively. Dim1 significantly separated LD and WD (CP and CPM), and Dim2 significantly separated WD and CP, CPM, CT, and LD. Among the metabolites with a principal component score > 0.5, Dim1 was mainly composed of phenolic acids, organic acids, and lipids, while Dim2 was mainly composed of phenolic acids and lipids, indicating that Dim1 and Dim2 significantly affected phenolic acids, organic acids, and lipids (Figure 4B).

#### 3.2.2. Difference analysis of metabolites between samples

**Figure 5:**
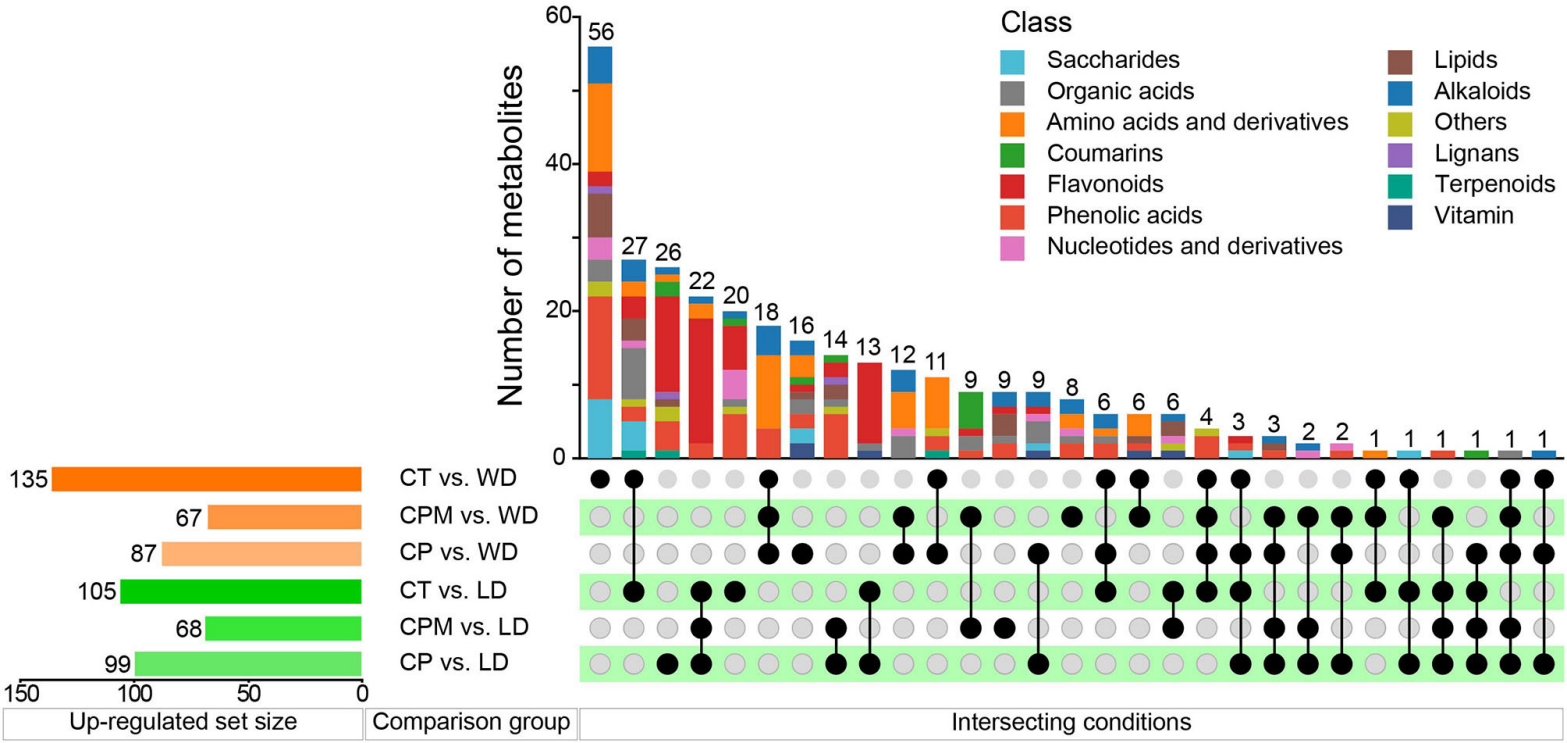
Differential metabolites upregulated by LD and WD between different comparison groups.

**Figure 6:**
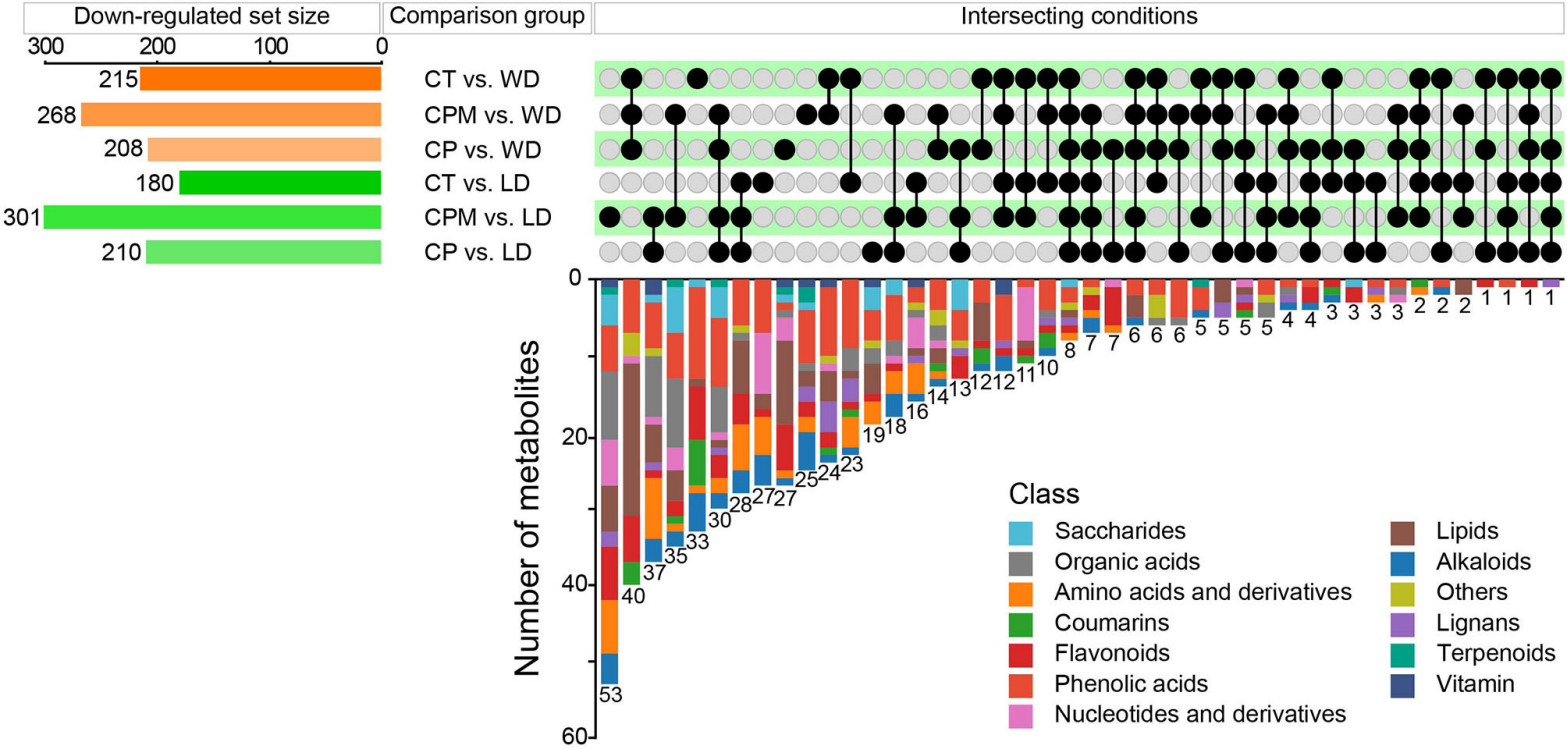
Differential metabolites downregulated by LD and WD between different comparison groups.

**Figure 7:**
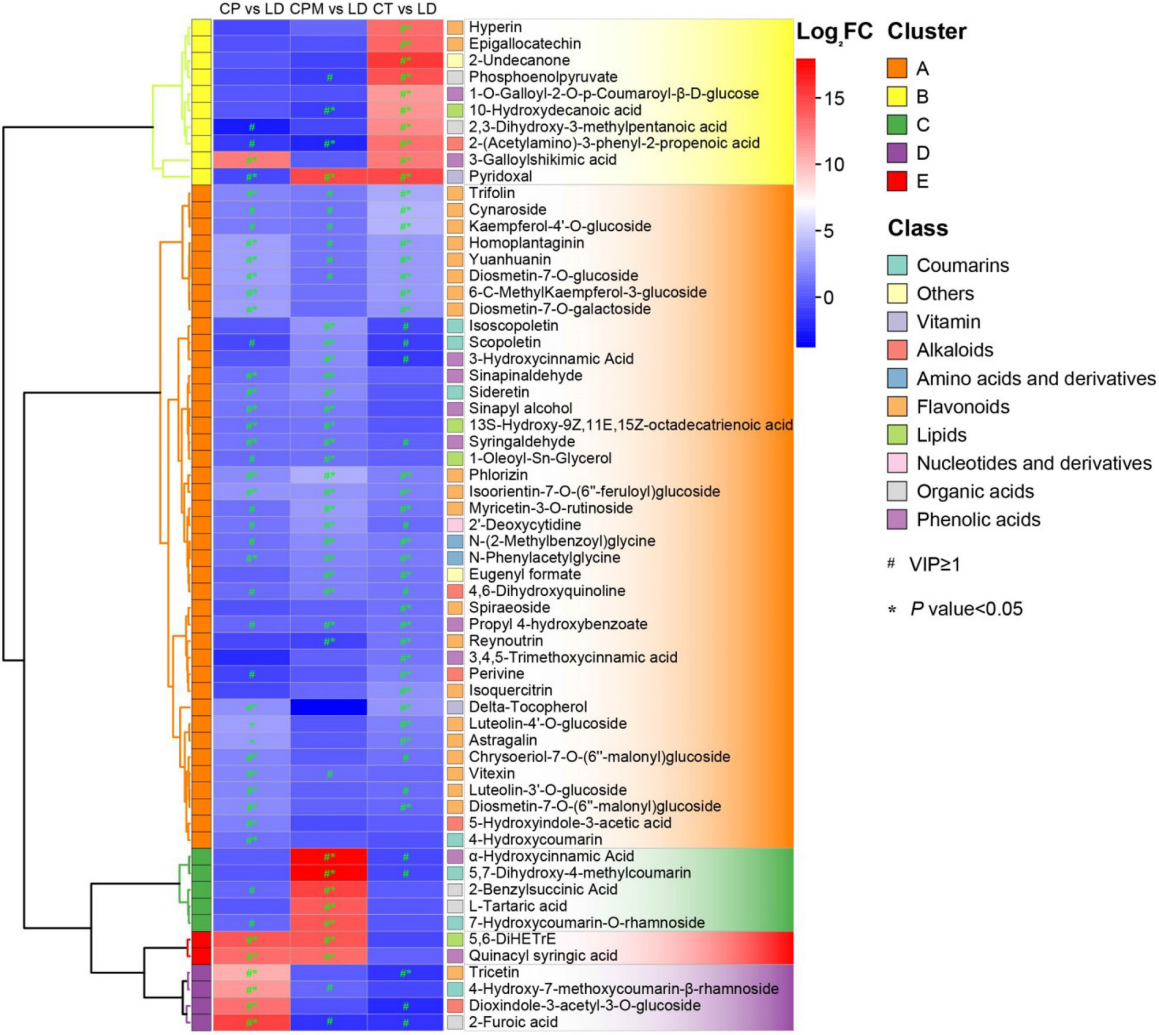
Hierarchical clustering heat map of LD dominant differential metabolites.

Differential metabolites were screened using | log_2_(fold change) |>1 and VIP≥1 as criteria. LD and WD were compared with CP, CPM, and CT in a two-by-two comparison to obtain the following six comparison groups of differential metabolites: CP vs. LD, CPM vs. LD, CT vs. LD, CP vs. WD, CPM vs. WD, and CT vs. WD, whose specific expression of DMs is shown in **Figures 6**, **7**. Compared with CP, CPM, and CT, 99, 68, and 105 metabolites were upregulated in LD, and 87, 67, and 135 metabolites were upregulated in WD. While LD upregulated metabolites were mainly concentrated in flavonoids, phenolic acids, alkaloids, and organic acids, WD upregulated metabolites were mainly concentrated in amino acids and derivatives, flavonoids, alkaloids, and organic acids (Figure 5). Previous studies showed that LD and WD had better food properties than CR from traditional production areas. It was speculated that the upregulation of the abovementioned DMs might be the reason based on our study. The comparative analysis found that there were 26, 9, 20, 16, 8, and 56 DMs upregulated only in CP vs. LD, CPM vs. LD, CT vs. LD, CP vs. WD, CPM vs. WD, and CT vs. WD, respectively. Including flavonoids (17 DMs), phenolic acids (3 DMs), amino acids and derivatives (2 DMs), alkaloids (1 DM), and coumarins (1 DM), 23 DMs were all upregulated in CP vs. LD, CPM vs. LD, and CT vs. LD. In the KEGG database, DMs were annotated to 7 metabolic pathways, mainly enriched to secondary metabolic biosynthesis (ko01110, 6 DMs) and flavonoid biosynthesis (ko00941, 6 DMs). Twenty-four DMs, including amino acids and derivatives (10), phenolic acids (7), alkaloids (4), others (1), and organic acids (1), were all upregulated in CP vs. WD, CPM vs. WD, and CT vs. WD. The KEGG database annotated 36 metabolic pathways, with the majority enriched to secondary metabolic biosynthesis (ko01110, 8 DMs) and metabolic pathways (ko01100, 13 DMs).

Compared with CP, CPM, and CT, 210, 301, and 180 metabolites were downregulated in LD, while 208, 268, and 215 metabolites were downregulated in WD (Figure 6). The comparative analysis found 19, 53, 27, 27, 25, and 33 DMs that were downregulated only in CP vs. LD, CPM vs. LD, CT vs. LD, CP vs. WD, CPM vs. WD, and CT vs. WD, respectively. Including phenolic acids (13 DMs), flavonoids (9 DMs), amino acids and derivatives (8 DMs), lipids (8 DMs), alkaloids (6 DMs), others (4 DMs), organic acids (3 DMs), lignans (2 DMs), and saccharides (1 DM), 54 DMs were all downregulated in CP vs. LD, CPM vs. LD, and CT vs. LD. In the KEGG database, DMs were annotated to 12 metabolic pathways, mainly enriched to metabolic pathways (ko01100, 5 DMs), tryptophan metabolism (ko00380, 3 DMs), and linoleic acid metabolism (ko00591, 3 DMs). Seventy-two DMs, including lipids (27 DMs), phenolic acids (14 DMs), flavonoids (8 DMs), others (7 DMs), coumarins (4 DMs), lignans (4 DMs), amino acids and derivatives (2 DMs), organic acids (2 DMs), alkaloids (2 DMs), saccharides (1 DM), and nucleotides and derivatives (1 DM) were all downregulated in CP vs. WD, CPM vs. WD, and CT vs. WD. The KEGG database annotated 15 metabolic pathways, with the majority enriched to secondary metabolic biosynthesis (ko01110, 9 DMs) and metabolic pathways (ko01100, 10 DMs).

#### 3.2.3. Screening for dominant differential metabolites

Screening for metabolites with a *p* < 0.05 and their upregulation in LD or WD on the basis of metabolite difference analysis were required to determine the dominant differential metabolites of the two species of CR from Guizhou. From these two processes, 28 dominant differential metabolites were found in CP vs. LD, including yuanhuanin, isoorientin-7-O-(6^′′^-feruloyl)glucoside, 4-Hydroxy-7-methoxycoumarin-β-rhamnoside, N-Phenylacetylglycine, and dioxindole-3-acetyl-3-O-glucoside; 25 prominent differential metabolites were detected in CP vs. LD, including 4,6-Dihydroxyquinoline, N-Phenylacetylglycine, 5,7-Dihydroxy-4-methylcoumarin, phlorizin, α-Hydroxycinnamic acid, and isoorientin-7-O-(6^′′^-feruloyl)glucoside; and 33 prominent differential metabolites were discovered in CT vs. LD, including isoorientin-7-O-(6^′′^-feruloyl)glucoside, 2-(Acetylamino)-3-phenyl-2-propenoic acid, N-Phenylacetylglycine, 3-Galloylshikimic acid, and phlorizin (Figure 7). A comprehensive investigation of three groups revealed that the three dominant differential metabolites, phlorizin, N-Phenylacetylglycine, and isoorientin-7-O-(6″-feruloyl) glucoside, were significantly upregulated in LD, with isoorientin-7-O-(6″-feruloyl) being detected in CR for the first time.

**Figure 8:**
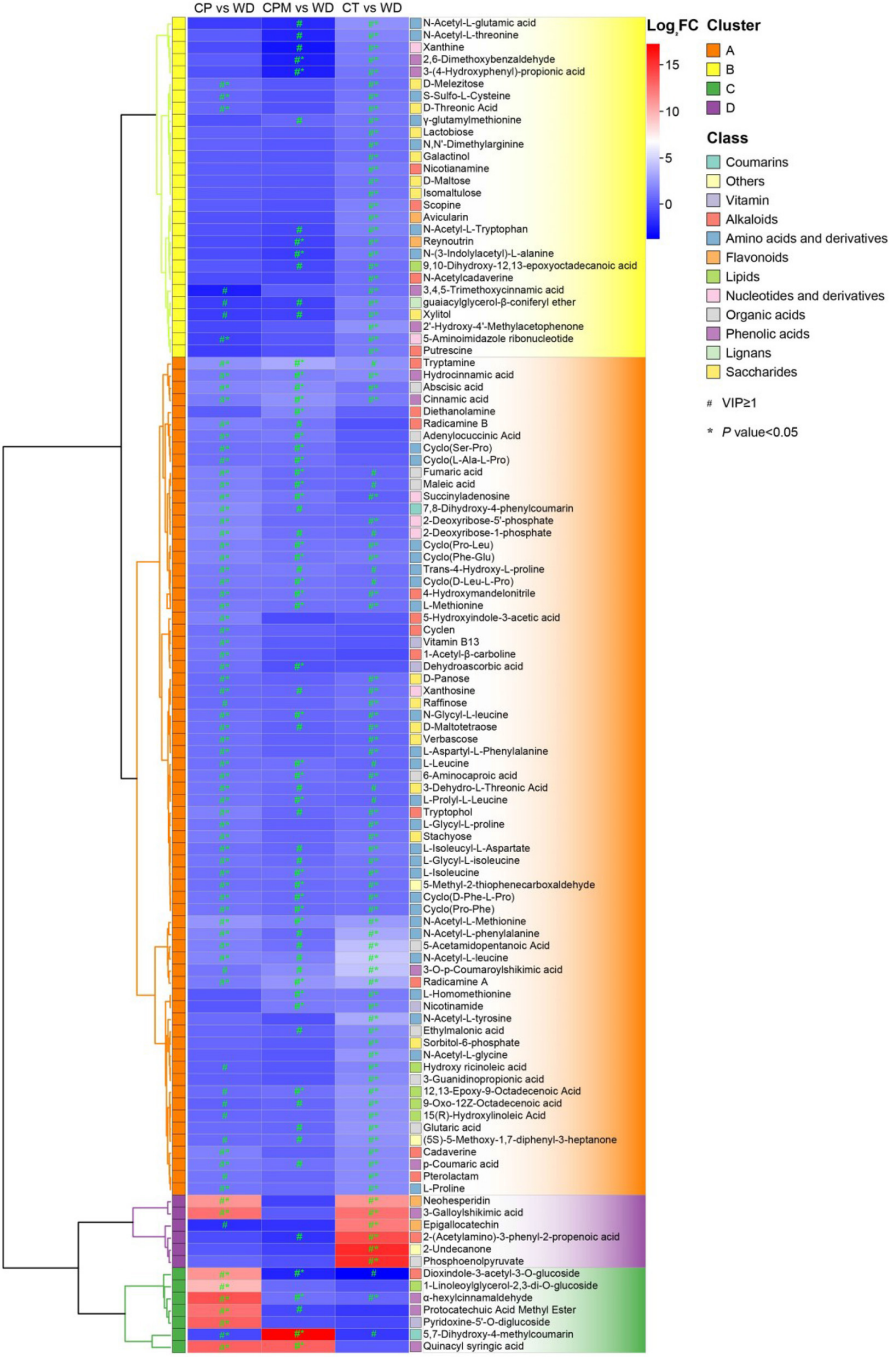
Hierarchical clustering heat map of WD dominant differential metabolites.

There were 57 dominant differential metabolites found in CP vs. WD, including dioxindole-3-acetyl-3-O-glucoside, N-Acetyl-L-Methionine, 7,8-Dihydroxy-4-phenylcoumarin, hesperetin-7-O-neohesperidoside(Neohesperidin), and alpha-hexylcinnamaldehyde. Approximately 25 prominent differential metabolites were detected in CPM vs. WD, including tryptamine, N-Acetyl-L-Methionine, 5,7-Dihydroxy-4-methylcoumarin, and abscisic acid. 2-(Acetylamino)-3-phenyl-2-propenoic acid^*^, N-Acetyl-L-leucine, epigallocatechin, 3-Galloylshikimic acid, and sorbitol-6-phosphate were among the 76 prominent differential metabolites discovered in CT vs. WD (Figure 8). A comprehensive investigation of 3 groups revealed that the 10 dominant metabolites, L-Methionine, hydrocinnamic acid, radicamine A, 4-Hydroxymandelonitrile, L-Isoleucine^*^, abscisic acid, N-Acetyl-L-Methionine, cinnamic acid, cyclo(Phe-Glu), and cyclo(Pro-Leu)^*^, were significantly upregulated in WD, with N-Formyl-L-Methionine, 4-Hydroxy-L-Isoleucine, cyclo(Phe-Glu), cyclo(Pro-Leu)^*^, cinnamic acid, hydrocinnamic acid, and 4-Hydroxymandelonitrile being among the seven prominent differential metabolites that were detected in CR for the first time.

### 3.3. Network pharmacology analysis

#### 3.3.1. Network pharmacological analysis of LD dominant differential metabolites

By estimating the appropriate target proteins of the two dominant differential metabolites in LD, a total of 196 targets were found. The protein-protein interaction (PPI) network was constructed using the 196 targets and visualized using Cytoscape (3.7.0, Supplementary Figure S1A). The targets with ≥10 degrees were HSP90AA1, ESR1, MAPK1, HSPA8, LCK, CREBBP, HRAS, HSPA1A, APP, MAP2K1, PPARA, HSPA5, PARP1, and PARP1, suggesting a more crucial function for the targets in the PPI network map.

**Figure 9:**
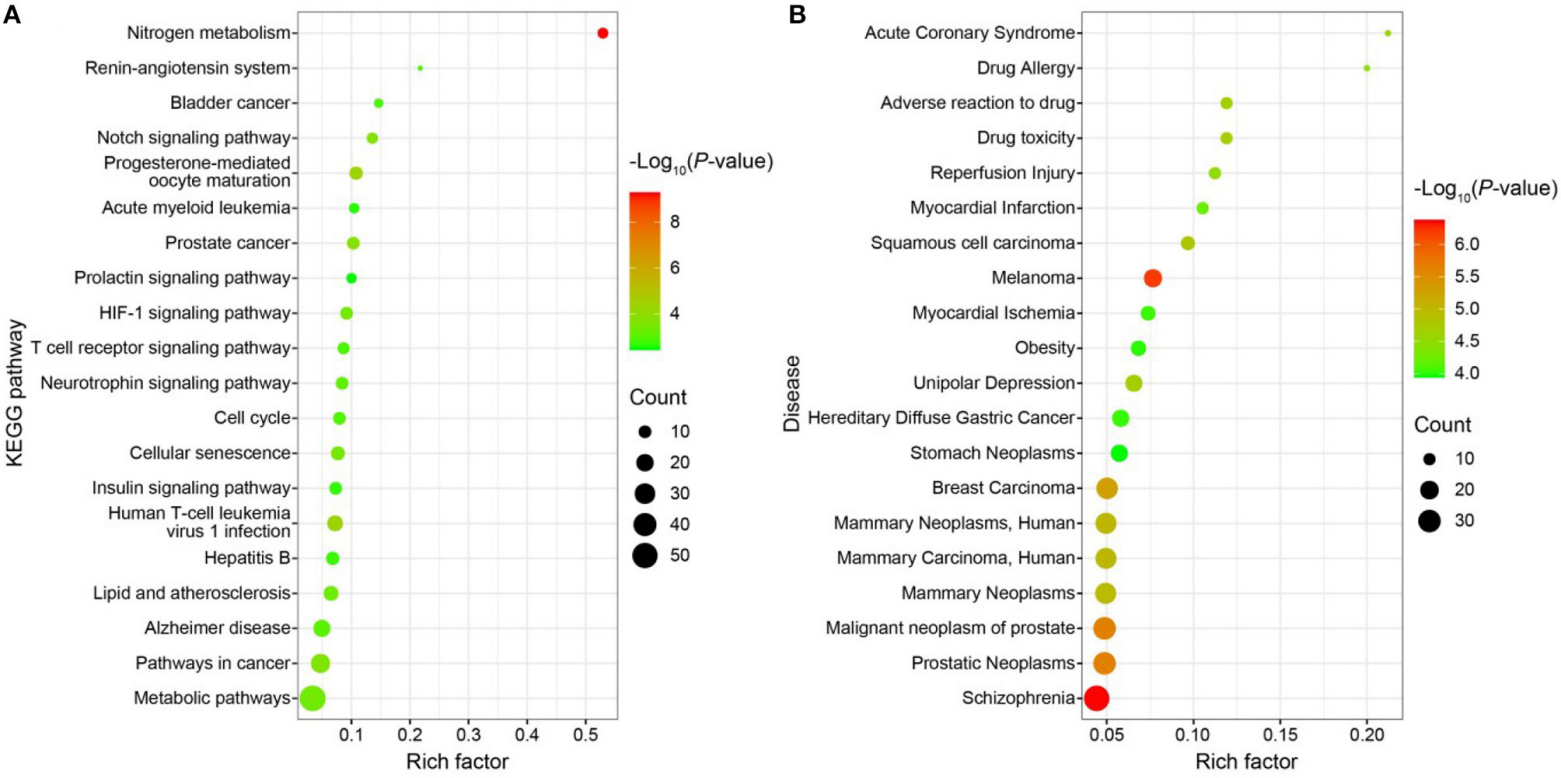
**(A)** Bubble map of KEGG enrichment pathways; **(B)** Bubble map of disease enrichment at the target of LD dominant chemistry.

Using the DAVIDv database (6.8, https://david.ncifcrf.gov/), GO functional enrichment analysis, KEGG pathway enrichment analysis, and disease enrichment analysis were carried out on the 196 targets. For the statistically significant GO functional analysis, KEGG pathway enrichment analysis, and disease enrichment analysis, a *p* < 0.01 was chosen. Approximately 169 items, including 98 BP, 32 CC, 39 MF, 25 KEGG pathway analysis entries (Figure 9A), and 87 disease enrichment analysis entries (Figure 9B), were derived from the GO functional analysis (Supplementary Figure S1B). The top 20 entries of each were selected and visualized using the bioinformatics website (http://www.bioinformatics.com.cn/). Histone H3-K9 demethylation, notch receptor processing, protein phosphorylation, one-carbon metabolic process, amyloid precursor protein catabolic process, among others, were BP's primary inclusions in the GO functional analysis. Extracellular MF mostly comprises histone demethylase activity, dioxygenase activity, zinc ion binding, carbonate dehydratase activity, and hydro-lyase activity. CC primarily includes cytosol, plasma membrane, an important component of the plasma membrane, gamma-secretase complex, and other cellular components. Nitrogen metabolism, progesterone-mediated oocyte maturation, human T-cell leukemia virus 1 infection, prostate cancer, notch signaling pathway are among the major topics covered by KEGG pathway analysis. The illness enrichment analysis revealed that oncological diseases, cardiovascular system diseases, neurological disorders, metabolic system diseases, and metabolic diseases accounted for the majority of the 196 targets matching LD-dominating differential metabolites. A “drug-component-target-network” map of LD was created using Cytoscape software (3.7.0) based on the screening of 2 components, 196 targets, and 20 pathways (Supplementary Figure S2A).

#### 3.3.2. Network pharmacological analysis of WD dominant differential metabolites

The target proteins corresponding to the other nine prominent differential metabolites in WD were predicted, and a total of 487 targets acting on the nine active components were discovered since the Canonical SMILES of cyclo (Phe-Glu) could not be accessed from the PubChem database. The PPI network map was visualized using Cytoscape (3.7.0), and it revealed that the targets with ≥50 degrees were SRC, MAPK1, HSP90AA1, CTNNB1, HDAC1, GRB2, RXRA, CREBBP, RELA, ESR1, MAPK14, and PTPN11. This suggests that the targets play a more crucial role in the PPI network map (Supplementary Figure S2B).

**Figure 10:**
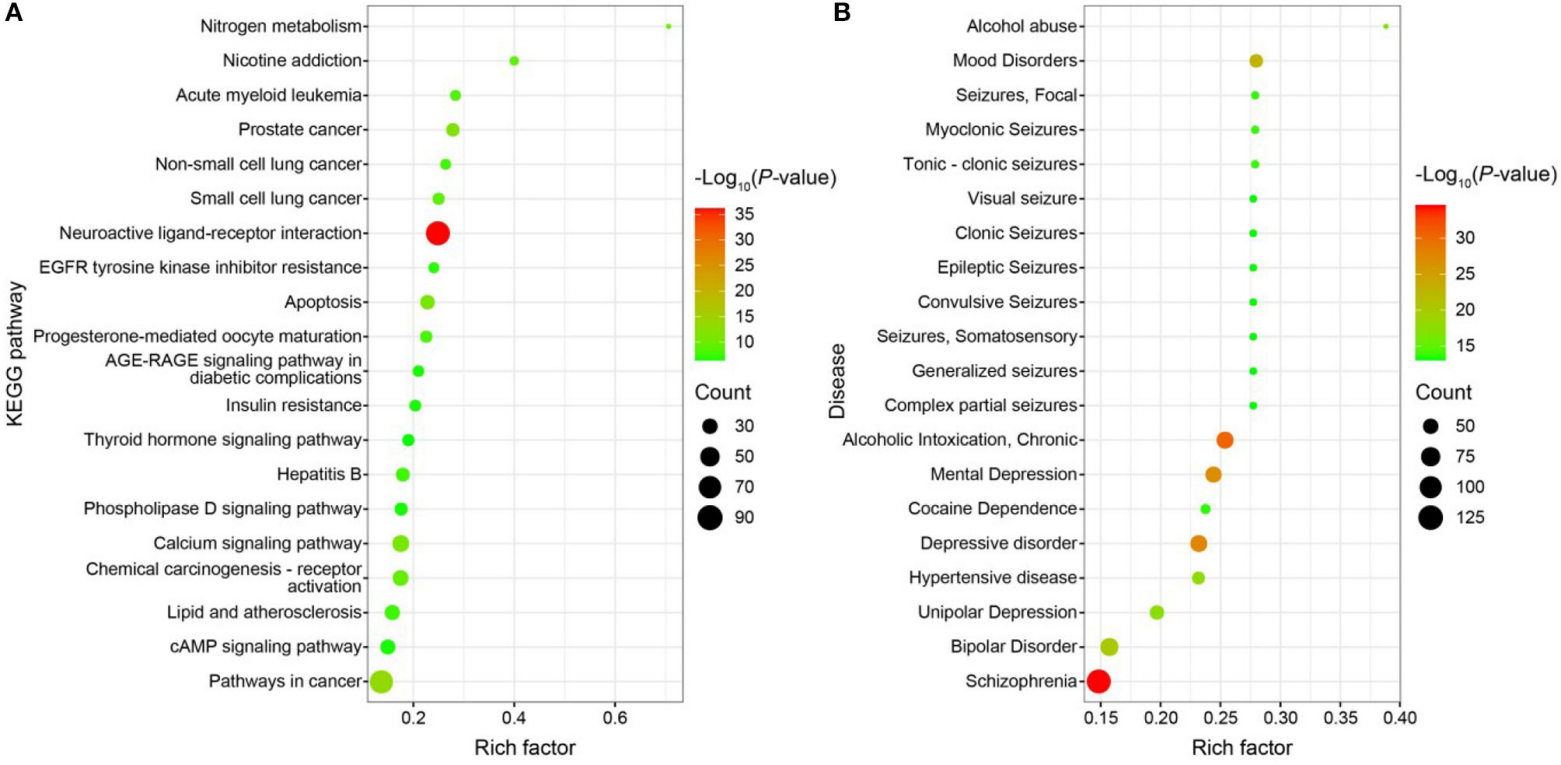
**(A)** Bubble map of KEGG enrichment pathways; **(B)** Bubble map of disease enrichment at the target of WD dominant chemistry.

GO functional enrichment, KEGG pathway enrichment, and disease enrichment were carried out on 487 targets using the DAVID database (6.8, https://david.ncifcrf.gov/). GO functional analysis, KEGG pathway analysis, and illness enrichment analysis were all chosen as statistically significant at a *p* < 0.01 each. Moreover, 645 GO functional analyses yielded a total of 645 entries (Supplementary Figure S3A), of which 407 were BP, 76 were CC, 162 were MF, 114 were KEGG pathway analyses (Figure 10A), and 361 were disease enrichment analyses (Figure 10B). The top 20 entries were chosen for bioinformatics visualization (http://www.bioinformatics.com.cn/). Chemical synaptic transmission, proteolysis, a reaction to lipopolysaccharide, an excitatory postsynaptic potential, a response to the medication, etc., were the primary components of BP in the GO functional analysis. CC mostly consists of a membrane raft, a plasma membrane, a presynaptic membrane, an integral component of the plasma membrane, and other membranes. The majority of the KEGG pathway analysis topics include neuroactive ligand-receptor interaction, cancer-related pathways, prostate cancer, apoptosis, calcium signaling pathways, etc. The 487 targets that corresponded to the major differential metabolites of WD were primarily associated with neurological and cardiovascular disorders, according to the disease enrichment analysis. The “drug-component-target-network” network map of WD was created using Cytoscape software (3.7.0) based on the nine components, 487 targets, and 20 pathways discovered during the screening (Supplementary Figure S3B).

Secondary metabolites from plants are a class of tiny molecules having a wide range of chemical structures and biological activity, as well as significant biological functions (29). In the plant world, flavonoids are extensively dispersed and have pharmacological actions, including anticancer, therapy for cardiovascular conditions, and hypoglycemia (40, 54, 55). Diabetes, cancer, bacterial infections, and neurological problems can all be treated with cinnamic acid and its phenolic acid metabolite derivatives (56, 57). An important family of chemical molecules known as alkaloids has a number of pharmacological properties, including analgesic, antibacterial, and anticancer properties (58, 59). The main differential metabolites of LD were flavonoids and amino acids, whereas those of WD were amino acids, alkaloids, phenolic acids, and organic acids, according to our screening of the prominent differential metabolites of LD and WD. By creating a “component-target-pathway” network topology, network pharmacology can graphically represent the efficacy of TCM through various components and targets, which is congruent with the holistic perspective and principles of diagnosis and treatment in TCM (60). We used network pharmacology to predict the pharmacological mechanisms of action of the dominant metabolites of LD and WD and discovered that the two dominant chemical components of LD may be closely related to nervous system protection, cardiovascular system improvement, oncological diseases, and diabetes treatment, while the nine dominant chemical components of WD may be closely related to nervous system protection and cardiovascular system improvement.

## Conclusions

In this study, with the results of plant traits and DNA barcoding molecular identification taken together, we believe that LD and WD are *C. tangshen* and *C. pilosula*, respectively, which are included in the Chinese Pharmacopeia. Our data revealed that LD and WD shared 1,054 (94.4%) metabolites with three species (*C. pilosula, C. pilosula* var. *modesta*, and *C. tangshen*) of CR from traditional production areas, with remarkably similar metabolite profiles. Therefore, we believe that LD and WD produced in Guizhou can be utilized medicinally and promoted. Furthermore, LD and WD each had 3 and 10 dominant differential metabolites, respectively, of which many were flavonoids and amino acids. The dominant metabolites were mainly enriched in secondary metabolic biosynthesis, metabolic pathways, flavonoid biosynthesis, tryptophan metabolism, and linoleic acid metabolism. Compared with the traditional CR production areas, LD and WD were influenced by the climate of Guizhou with low latitude, low latitude, high altitude, high precipitation, high average annual temperature, and relative humidity. They were more prone to accumulating some amino acids, phenolic acids, and organic acids, which may be the reason for their excellent food attributes. The metabolites isoorientin-7-O-(6″-feruloyl) glucoside, N-formyl-L-methionine, 4-hydroxy-L-isoleucine, cyclo (Phe-Glu), cyclo (Pro-Leu)^*^, cinnamic acid, hydrocinnamic acid, and 4-hydroxymandelonitrile were detected in CR for the first time in our study. The network pharmacological analysis found that the two dominant differential metabolites of LD might be related to protecting the nervous system, improving the cardiovascular system, treating tumor diseases, and managing diabetes. The nine dominant differential metabolites of WD might be related to protecting the nervous system and curing cardiovascular diseases. This study provided a scientific foundation for the medical use and promotion of LD and WD, as well as a reference for CR quality control, resource exploitation, and novel drug development.

## Data availability statement

The original contributions presented in the study are included in the article/Supplementary material. The data presented in the study are deposited in the ProteomeXchange Consortium, accession number PXD044874.

## Author contributions

KZ: Validation, Writing—original draft, Investigation, Methodology. DZ: Validation, Writing—original draft, Conceptualization. QY: Resources, Writing—review and editing. LL: Writing—original draft. JX: Writing—review and editing. YW: Writing—review and editing. QY: Writing—review and editing. FW: Conceptualization, Funding acquisition, Writing—original draft. SL: Investigation, Methodology, Resources, Writing—original draft.
