# Supplementary figures and images for: Integrated widely targeted metabolomics and network pharmacology revealed quality disparities between Guizhou and conventional producing areas of Codonopsis Radix
Source: Front Nutr. 2023 Oct 17;10:1271817. doi: 10.3389/fnut.2023.1271817 (PMC10616484; doi:10.3389/fnut.2023.1271817)

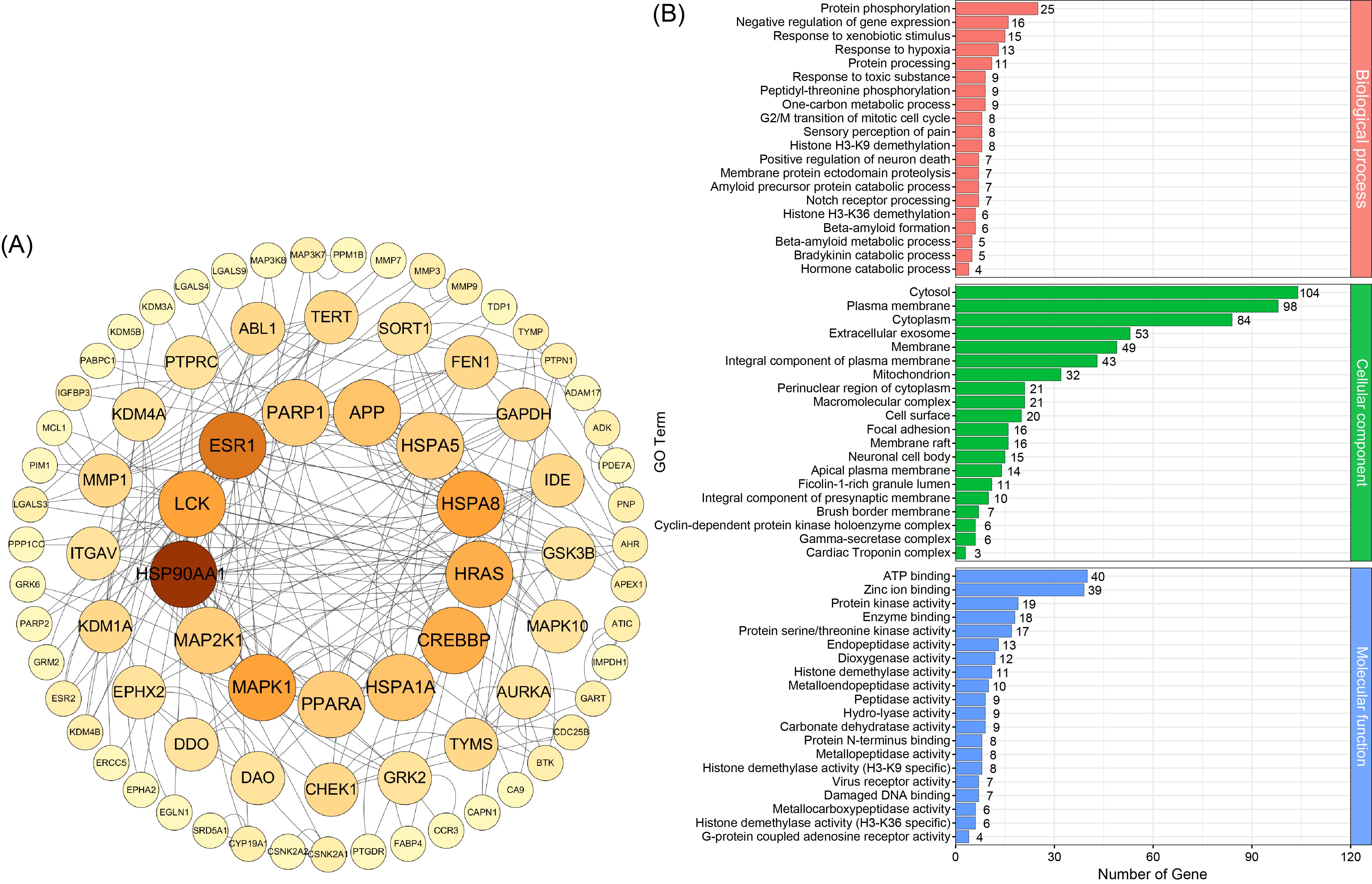

Supplement: Supplementary file 2 [file Image_1.jpg]

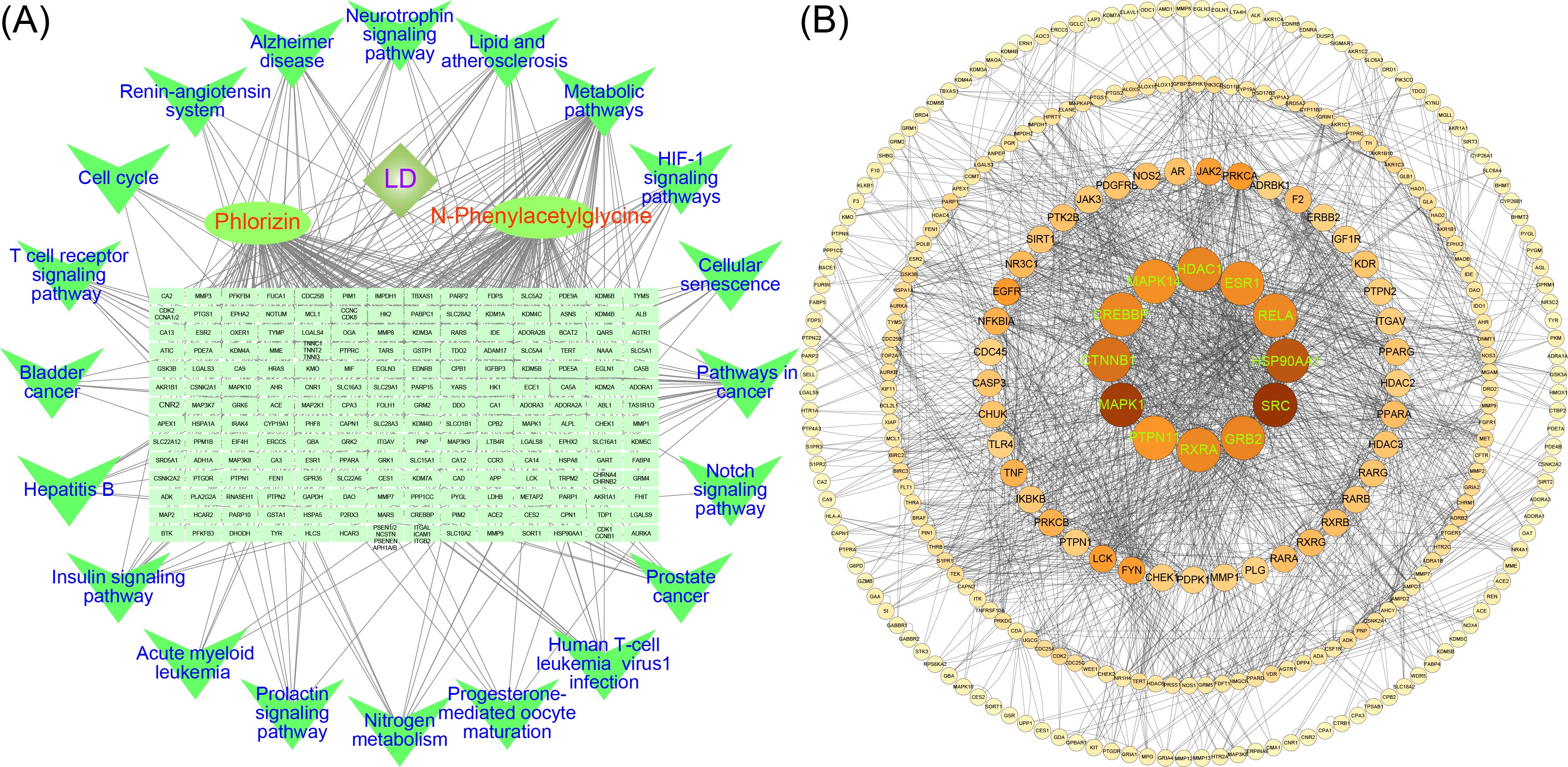

Supplement: Supplementary file 3 [file Image_2.jpg]

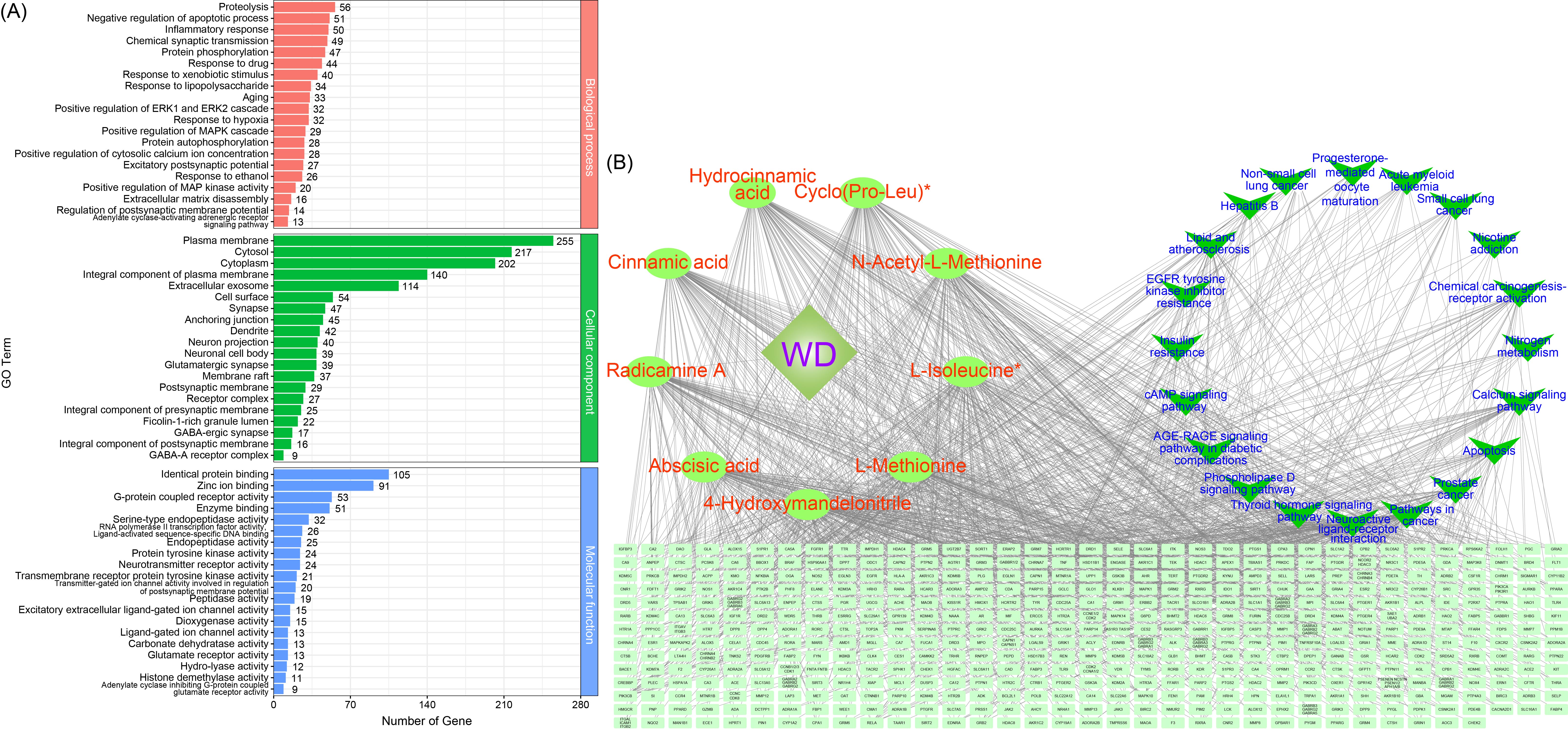

Supplement: Supplementary file 4 [file Image_3.jpg]
